# A CD276-Targeted Antibody-Drug Conjugate to Treat Non-Small Lung Cancer (NSCLC)

**DOI:** 10.3390/cells12192393

**Published:** 2023-09-30

**Authors:** Jiashuai Zhang, Zhuoxin (Zora) Zhou, Kai Chen, Seulhee Kim, Irene Soohyun Cho, Tanvi Varadkar, Hailey Baker, Ju Hwan Cho, Lufang Zhou, Xiaoguang (Margaret) Liu

**Affiliations:** 1 Department of Biomedical Engineering, The Ohio State University (OSU), 151 West Woodruff Ave, Columbus, OH 43210, USA; zhang.13284@osu.edu (J.Z.); kim.8819@osu.edu (S.K.); baker.2718@osu.edu (H.B.); zhou.4212@osu.edu (L.Z.); 2 Department of Chemical and Biomolecular Engineering, The Ohio State University (OSU), 151 W Woodruff Ave, Columbus, OH 43210, USA; zhou.4213@osu.edu (Z.Z.); chen.12100@osu.edu (K.C.); cho.766@osu.edu (I.S.C.); varadkar.1@osu.edu (T.V.); 3 Comprehensive Cancer Center, The Ohio State University (OSU), 460 West 10th Avenue, Columbus, OH 43210, USA; cho.622@osu.edu

**Keywords:** non-small cell lung cancer, CD276-targeted therapy, antibody-drug conjugate

## Abstract

Non-small cell lung cancer (NSCLC) patients, accounting for approximately 85% of lung cancer cases, are usually diagnosed in advanced stages. Traditional surgical resection and radiotherapy have very limited clinical benefits. The objective of this study was to develop and evaluate a targeted therapy, antibody-drug conjugate (ADC), for NSCLC treatment. Specifically, the CD276 receptor was evaluated and confirmed as an ideal surface target of NSCLC in the immunohistochemistry (IHC) staining of seventy-three patient tumor microarrays and western blotting analysis of eight cell lines. Our anti-CD276 monoclonal antibody (mAb) with cross-activity to both human and mouse receptors showed high surface binding, effective drug delivery and tumor-specific targeting in flow cytometry, confocal microscopy, and in vivo imaging system analysis. The ADC constructed with our CD276 mAb and payload monomethyl auristatin F (MMAF) showed high anti-NSCLC cytotoxicity to multiple lines and effective anti-tumor efficacy in both immunocompromised and immunocompetent NSCLC xenograft mouse models. The brief mechanism study revealed the integration of cell proliferation inhibition and immune cell reactivation in tumor microenvironments. The toxicity study did not detect off-target immune toxicity or peripheral toxicity. Altogether, this study suggested that anti-CD276 ADC could be a promising candidate for NSCLC treatment.

## 1. Introduction

Lung cancer, representing a significant global health challenge, is one of the most prevalent cancers and a leading cause of cancer-related deaths [1]. Among the two major subtypes, non-small cell lung cancer (NSCLC) accounts for approximately 80–85% of lung cancers, including adenocarcinoma (AD, 40–50%), squamous cell carcinoma (SqCC, 20–30%), and large cell carcinoma (LCC, ~10%) [1,2,3]. Most NSCLC patients are diagnosed at advanced or metastatic stages III or IV, which limits the surgical treatment option [4].

Over the years, significant progress has been made in the treatment of NSCLC, with the incorporation of traditional radiotherapies, chemotherapies inhibiting cell division and DNA replication with limited clinical benefits, and targeted therapies dealing with certain gene mutations [3,5]. As reviewed in the literature [3,6] or described on the website of U.S. Food and Drug Administration (FDA) list of 84 approved NSCLC therapies, tyrosine kinase inhibitors gefitinib, erlotinib and novel drugs targeting mutant epidermal growth factor receptor (EGFR) [7], enhertu (trastuzumab-deruxtecan) targeting epidermal growth factor receptor 2 (HER2) mutation [8,9], xalkori (crizotinib), zykadia (ceritinib), and alectinib targeting gene rearrangement in echinoderm microtubule-associated protein-like 4 (EML4) and anaplastic lymphoma kinase (ALK), mekinist (trametinib) targeting Kirsten rat sarcoma viral oncogene (KRAS) with mutation [3,10], and rybrevant (bispecific amivantamab) targeting EGFR and MET have been established for subtype AD treatment. Nintedanib and dovitinib targeting the amplification of fibroblast growth factor receptor 1 (FGFR1), BKM120, PX-866 and GDC-0941 targeting phosphatidylinositol-4,5-bisphosphate 3-kinase catalytic subunit alpha (PIK3CA) with mutation, and MK-2206 targeting AKT in subtype SqCC have been investigated [11,12]. The cyclin-dependent kinase (CDK) inhibitors targeting CDKNA2/p16INK4, cabozantinib, and selumetinib targeting tumor suppressor NF1 mutations, and TP53-MDM2 inhibitors have been investigated for the treatment of both AD and SqCC. Despite the improved median overall survival, disease-free survival, and overall response rate of these targeted therapies, the 5-year survival rates of NSCLC patients post-treatment are still low, e.g., 24–33% [4,13,14].

Recently, the FDA approved immune checkpoint blockers (ICBs) [3,6], such as anti-PD-1 (programmed cell death protein 1) nivolumab and pembrolizumab, anti-PD-L1 (programmed cell death ligand 1) atezolizumab and durvalumab, anti-cytotoxic T cell antigen 4 (CTLA-4) tremelimumab-actl, and other inhibitors [15,16,17], which have been investigated for NSCLC treatment, either as monotherapies or in combination with other therapies [3]. The anti-cancer mechanism of IBCs reactivates the cytotoxicity of CD8^+^ T cells and enhance the anti-tumor immune response in the tumor microenvironment (TME) by blocking the PD-L1/PD-1 signaling pathway [12,13,14,18,19]. In addition to PD-1 and PD-L1, CD276 (also known as B7-H3) serves as a potential immune checkpoint molecule and highly expressed in various solid tumors and exhibits limited expression in normal tissues [19,20]. Moreover, CD276 is also involved in tumor progression, metastasis, and poor clinical outcomes in various malignancies [21,22]. As an immune-regulating member of the B7 family, a tumor cell surface molecule, and a tumor vessel endothelial marker [23,24,25], CD276 has emerged as a potential target for NSCLC therapy, either alone or in combination with PD-1 [26,27,28].

The objectives of this study were to develop and evaluate the CD276-targeting antibody-drug conjugate (ADC) for NSCLC treatment. The high surface expression of CD276 was confirmed in NSCLC cell lines and patient tissues. ADC was constructed using our murine anti-CD276 monoclonal antibody (mAb) and microtubule depolarizing payload monomethyl auristatin F (MMAF). The in vitro evaluations showed high surface binding, drug delivery, and cytotoxicity to NSCLC lines. Further, in vivo studies demonstrated effective anti-NSCLC efficacy in both immunocompetent and immunocompromised xenograft mouse models. In addition to cell proliferation inhibition and apoptosis, tumoral immunity was also detected in the NSCLC tissues post-ADC treatment. This study suggested that CD276 targeting therapy is a promising syngenetic therapy to treat NSCLC.

## 2. Materials and Methods

### 2.1. Ethics Statement

The animal studies were conducted according to the Institutional Animal Care and Use Committee (IACUC) Protocol 2022A00000071, which was approved by the Institutional Biosafety Committee of Ohio State University (OSU).

### 2.2. Cell Lines and Culture Media

The human NSCLC cell lines, H-460 (Perkinelmer, Waltham, MA, USA), H460-FLuc (Perkinelmer), and H358 (American Type Culture Collection [ATCC], Manassas, VA, USA) were cultivated in an RPMI-1640 medium (Gibco, Grand Island, NY, USA) supplemented with 10% heat-inactivated fetal bovine serum (HI FBS, *v*/*v*; Gibco) and 1% penicillin streptomycin (P/S, *v*/*v*). The mouse Lewis Lung Cancer cell line LLC-Fluc (ATCC) was cultivated in a DMEM medium supplemented with 10% HI FBS and 1% P/S. The seed culture of hybridoma-producing murine anti-CD276 mAb was maintained in a serum-free medium and Hybridoma-SFM supplemented with 4 g/L of glucose and 4 mM of L-glutamine in 125 or 250 mL shaker flasks at 130 rpm or 250 mL spinner flasks at 175 rpm. All these cell lines were incubated in a humidified CellXpert C170 CO_2_ incubator (Eppendorf North America, Enfield, CT, USA) with a set point of 37 °C and 5% CO_2_. All the cell culture media, medium supplements, and bioreagents used in this project were purchased from Gibco or Fisher Scientific (Waltham, MA, USA) unless otherwise specified.

### 2.3. NSCLC Patient Tissue Microarray and Immunohistochemistry (IHC) Staining

The patient tissue microarray slide, including 73 cores of NSCLC patient tissue microarrays (TMAs) with duplication (LC1461), was purchased from US Biomax (Derwood, MD, USA). The TMA slides were processed following the procedure provided by the biomanufacturer and stained with recombinant rabbit anti-mouse/human CD276 antibody (AB134161, Abcam, Cambridge, UK) and horseradish peroxidase (HRP)-conjugated secondary anti-rabbit antibody (Cell Signaling Technology, Danvers, MA, USA) to detect CD276 expression. The positive staining was visualized with DAB (3,3′-Diaminobenzidine) and imaged with a Vectra 3 automated quantitative pathology imaging system (Akoya Biosciences, Marlborough, MA, USA). Following our previously established TMA image analysis [29,30], we used ImageJ software (Version 1.53t, National Institutes of Health, Bethesda, MD, USA) to quantitatively score CD276 expression with the Plugins-Analyze-RGB Measure. The expression score was calculated as red intensity/blue intensity with criteria of >1.1 as high and medium expression and <1.1 as low or no expression.

### 2.4. Anti-CD276 mAb Production and Purification

The murine anti-CD276 mAb-producing hybridoma cells were cultivated in the Hybridoma-SFM medium (Gibco, Grand Island, NY, USA) supplemented with 4 mM L-glutamine and 4 g/L glucose and 1% P/S (*v*/*v*). The 30 or 60 mL cultures in SF125 or SF250 shaker flasks were seeded at a viable cell density (VCD) of 0.2 × 10^6^ cells/mL and cultivated at an agitation speed of 130 rpm. Fed-batch production was performed by feeding 4 mM L-glutamine, 4 g/L glucose, and 3.5 g/L Cell Boost #6 on days 3 and 5. CD276 mAb was harvested from supernatant when the cell viability was lower than 50% and purified using NGC liquid chromatography equipped with protein A column [12,29,31,32]. The purified mAb was desalted with 10 kDa MWCO Slide-A-Lyzer Dialysis Cassettes and concentrated with a 10 kDa MWCO concentrator.

### 2.5. Anti-CD276 mAb-MMAF Conjugation and Characterization

Following our established ADC construction protocol with optimization [12,29,31,33], the purified anti-CD276 mAb was diluted to a final concentration of 5 mg/mL in PBS and warmed to 37 °C in a heat block. A 5 mM of tris (2-carboxyethyl) phosphine (TCEP) solution was freshly prepared, and 44 molar equivalents (i.e., TCEP: mAb = 44) were added to the mAb solution. The mAb reduction reaction mixture was incubated at 37 °C in a heat block for 30 min. Then, seven molar equivalents of dibromomaleimide (DBM)-MMAF (a customer-designed product by MedChemExpress, Monmouth Junction, NJ, USA) from 10 mM stock was added to the TCPE reduced CD276 mAb, and the reaction mixture was gently mixed at room temperature for 1 h. About 100 µL of crude ADC was characterized using high-performance liquid chromatography (HPLC, Shimadzu, Columbia, MD, USA) equipped with an MABPac HIC-butyl column to confirm the conjugation rate of >95% and analyze the drug–antibody ratio (DAR). The synthesized ADC was purified and concentrated using a 10 kDa MWCO concentrator to remove excess small MW reagents and stored at −80 °C.

### 2.6. Flow Cytometry Analysis

The cell surface-binding rates of CD276 mAb to H460-FLuc, LLC-FLuc, and H358 cells were detected using a BD LSRII flow cytometer (BD Biosciences, San Jose, CA, USA). Briefly, CD276 mAb was labeled with an Alexa Fluor™ 647 (Invitrogen, Carlsbad, CA, USA) kit to generate CD276 mAb-AF647. About 1 × 10^6^ human NSCLC cells (H460-Fluc, H358, or LLC-Fluc) were suspended in a 1 mL flow cytometry buffer (1% FBS in PBS, *v*/*v*); then, 1 µg (for H460-FLuc, H358) or 5 µg (for LLC-FLuc) of CD276 mAb-AF647 was added, followed with incubation at room temperature for 30 min or 2 h, respectively, in the dark. Then, the reaction mixture was washed with PBS twice before applying it to a flow cytometer. A uniform cell suspension of 20,000 H460-FLuc or H358 cells and 10,000 LLC-FLuc cells were applied for surface binding analysis.

### 2.7. Western Blotting

The expressions of CD276 in normal human lungs, human NSCLC cell lines, and human NSCLC cell lines were also analyzed by western blotting. Specifically, cells were washed three times with cold PBS and lysed using a RIPA buffer to extract whole cellular proteins. Protein concentration was quantified using the bicinchoninic acid (BCA) method with a Pierce protein assay kit (Pierce, Cambridge, NJ, USA). Next, a total of 30 µg of proteins were loaded per lane onto a gradient SDS-PAGE, NuPAGE 4–12% gradient gel (Invitrogen), along with a protein size marker, Bio-Rad Precision Plus (Bio-Rad Laboratories, CA, USA), to separate proteins by molecular weight. The separated proteins were then transferred onto a PVDF membrane using a Bio-Rad power supply (Bio-Rad Laboratories) at a constant voltage of 100 V for 90 min. After transfer, the PVDF membrane was blocked with 5% non-fat milk in a TBST buffer and agitated at room temperature for 1 h. The primary antibodies of CD276 (Dilution 1:1000, AB134161) and β-actin (1:2000, sc-47778) were purchased from Abcam (Cambridge, UK) and Santa Cruz Antibodies (Santa Cruz, CA, USA), respectively. The membrane was then incubated at 4 °C with continuous agitation overnight. On the following day, the primary antibodies were discarded, and the membrane was washed three times with a TBST buffer on a shaker, with each wash lasting 5, 5, and 10 min, respectively. After the washing steps, horseradish peroxidase (HRP)-conjugated secondary antibodies (dilution 1:2000) specific to the mouse or rabbit from CST (Danvers, MA, USA) in 3% non-fat milk were applied to the membrane at room temperature for 1 h. After discarding the secondary antibody, the membrane was washed three times with TBST for 5, 5, and 10 min, respectively. Finally, the protein bands were visualized and quantified using an Odyssey Fc imaging system (LI-COR Biosciences, NE, USA). The expression of CD276 in TNBC cells was compared to the internal control, β-actin.

### 2.8. Live-Cell Confocal Microscopy

We investigated the surface binding and internalization of our anti-CD276 mAb using live-cell confocal imaging following established protocols in our previous publications [29,30,33]. H460-Fluc and human NSCLC were cultured in 35 mm glass bottom dishes (Cellvis, Mountain View, CA, USA) at a density of 1 × 10^4^ cells per dish in 1.5 mL of medium. To visualize the cytoplasm and nucleus, BacMam GFP Transduction Control (Invitrogen) and NucBlue™ Live ReadyProbes™ Reagent (Invitrogen), respectively, were used for staining following manufacturing protocols. Next, the AF647-labeled CD276 mAb was added to the cells at a final concentration of 1 or 5 µg per mL. Live-cell images were captured at 0–24 h after the addition of CD276 mAb-AF647 using a Nikon A1R-HD25 confocal microscope (Nikon USA, Melville, NY, USA). This experimental approach allowed us to dynamically monitor the binding and internalization of CD276 mAb into NSCLC over different time points.

### 2.9. In Vitro Cytotoxicity Assay

The cytotoxicity assay was performed using human NSCLC cell line H460-FLuc and mouse LLC-FLuc. With different cell division rates, 3000 H460-FLuc cells or 1000 LLC-FLuc cells with 200 uL of RPMI-1640 or a DMEM complete growth medium were seeded into each well in the 96-well plates. The cells were incubated at 37 °C in a CO_2_ incubator for 24 h, followed by the addition of free MMAF or ADC at dosages of 0, 10, 20, 40, 80, 120, 160, and 200 nM. The 3-(4,5-Dimethylthiazol-2-yl)-2,5-Diphenyltetrazolium Bromide Cell Proliferation Assay (MTT Assay) was performed following the biomanufacturer procedure after 5-day treatment. At the end of treatment, the spent medium was removed and replaced with 100 µL of the fresh culture medium supplemented with 10 µL of the MTT reagent in each well. After 2 h of incubation in the dark, 100 µL of the detergent reagent was added to each well until a purple color was developed. The OD value was read at 590 nm using a SpectraMax iD3 plate reader (San Jose, CA, USA), and IC_50_ values were calculated.

### 2.10. In Vivo Imaging System (IVIS)

The navigation of our anti-CD276 mAb to human NSCLC H460-FLuc xenograft tumor and mouse LLC-FLuc allograft tumor was monitored by detecting bioluminescent signal (tumor) and fluorescence signal (mAb) through IVIS Lumina Series III. The mice inoculated with FLuc-labeled NSCLC cells were intravenously (i.v.) injected with 50 µg of Cy5.5-labeled CD276 mAb. At 24 h post-mAb injection, 100 µL of luciferin (30 mg/mL stock) was intraperitoneally (i.p.) injected. After 10 min, the live mice were imaged under the IVIS machine for both bioluminescence at a wavelength of 550 nm and the targeted Cy5.5-labeled CD276 mAb at a wavelength of 750 nm. The major organs (liver, lung, heart, kidney, spleen, brain) and tumor were harvested to collect ex vivo images to confirm the tumor-specific targeting of CD276 mAb.

### 2.11. Whole Blood Analysis

At the end of the in vivo treatment, we collected mice blood for whole blood analysis after isoflurane was administrated to deeply anesthetize the mice. The cardiac puncture using a 39-gauge syringe was performed to obtain the largest volume of mice blood. We collected the blood, aliquoted it in two EDTA tubes, and immediately ran a whole blood analysis. For the remaining blood sample, we centrifuged and kept the serum samples at −80 °C for longer storage.

### 2.12. NSCLC Xenograft Mouse Models and In Vivo Treatment

The in vivo NSCLC treatments were tested in two mice models (immunocompetent and immunocompromised). The allograft C57BL/6 black mice were subcutaneously (s.c.) injected with 1 × 10^6^ of LLC-Fluc cells in each mouse when they were about 6 weeks old. Given the fast growth rate of LLC-Fluc, we started the i.v. administration of ADC at dosages of 12 mg/kg, 24 mg/kg, mAb only at a dosage of 12 mg/kg, or saline (control) two days after tumor cell implantation. The 9-week-old xenograft immunodeficient nude mice were s.c. injected with 3 × 10^6^ cells H460-FLuc cells each mouse. The i.v. administration of ADC or the control was initiated when tumor volume reached ~25 mm^3^, and the same dosage was given to C57BL/6 black mice. The C57BL/6 black mice and nude mice were treated twice a week until the biggest tumor volume reached >1500 mm^3^ and 1000 mm^3^, respectively. Mice’s body weight and tumor volume were measured twice a week during treatment.

### 2.13. Parafilm Section and Hematoxylin and Eosin (H&E) Staining

For paraffin section preparation, lung cancer tissue samples were fixed in 4% formalin for >24 h. After fixation, the samples were dehydrated through graded ethanol solutions and embedded in paraffin blocks. Sections of 4–5 μm thickness were cut from the paraffin blocks using a microtome and mounted onto glass slides. Subsequently, the sections were deparaffinized and rehydrated for staining. H&E staining was performed to visualize the cellular and tissue structures. The deparaffinized sections were incubated in a hematoxylin solution to stain the nuclei, followed by a brief rinse in running water. Next, the sections were differentiated in acid alcohol to remove excess hematoxylin. After rinsing, the sections were counterstained with an eosin solution, highlighting the cytoplasm and extracellular components. The stained sections were dehydrated using ethanol, cleared with xylene, and mounted with a coverslip using a mounting medium.

For H&E-stained sections, the assessment of cellular morphology and tissue architecture was carried out using an Eco microscope at 40× magnification. The characteristics of individual cells, including their size, shape, and staining patterns, as well as the overall arrangement and integrity of the tissue, were analyzed.

### 2.14. Statistical Analysis

All the in vitro experiments were performed with duplication, and the data were reported as mean ± standard error of the mean. A two-tailed *t*-test was used to analyze the significance of differences among groups, and the difference between the two groups was analyzed using one-way ANOVA. A statistical analysis was performed using GraphPad Prism software (version 10.0.3, Boston, MA, USA), and a *p*-value of <0.05 was used for all in vivo studies.

## 3. Results and Discussion

This study evaluated the surface receptor CD276 as a therapeutic target of NSCLC and developed an anti-CD276 mAb-derived ADC, i.e., CD276 mAb-MMAF, which integrates the anti-cancer mechanism of blocking the immune checkpoint, reactivating immune cells in tumor microenvironments, and inhibiting microtubule polymerization, to treat NSCLC.

### 3.1. CD276 Expression in NSCLC

**Figure 1:**
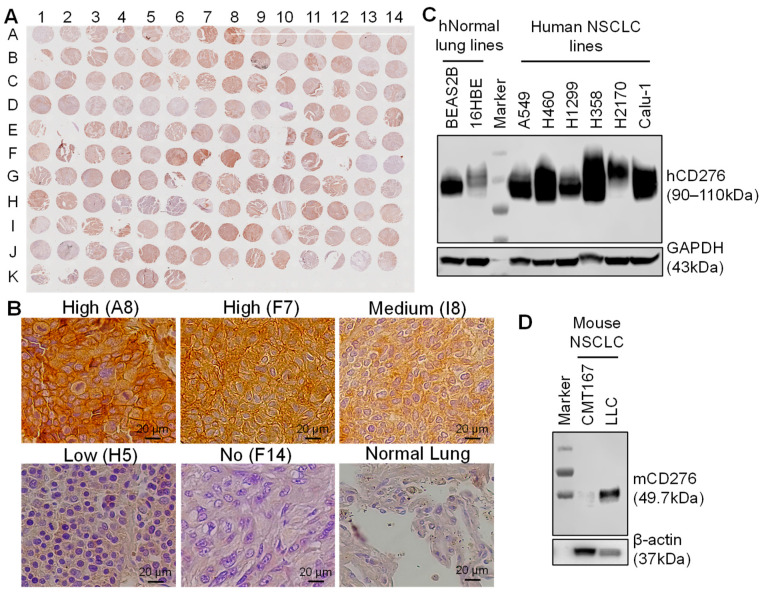
Evaluations of CD276 surface receptor in NSCLC lung cancers. (**A**) IHC staining of NSCLC TMA with anti-CD276 antibody. (**B**) Representative NSCLC patient tissues with high, medium, low, and no surface CD276 expression, with normal lung tissue as the control. (**C**) Western blotting of human NSCLC lines. (**D**) Western blotting of mouse NSCLC lines.

To assess the expression of CD276 receptor in NSCLC, the parafilm sectioned TMA slides with 73 cores (*n* = 2), including stages of IB, IIA/B, and IIIA of subtypes ADC, SqCC, and other carcinoma, and normal lung tissue (control, *n* = 2), were stained with anti-human CD276 mAb. The IHC images of NSCLC cores described in Figure 1A were analyzed using ImageJ to quantify and calculate the relative expression levels of CD276. The CD276 expression scores of most NSCLC tissues were higher than the normal lung tissues. It is found that 108 and 19 of 146 cores had high and medium expression (score of >1.0), respectively, and 15 cores had low or minimal expression (score of <1.0), with representative images described in Figure 1B. The IHC staining of TMA indicated that CD276 is a good target for treatment, which could cover approximately 74% of NSCLC patients.

Western blotting of the whole cellular proteins demonstrated an overexpression of receptor CD276 in multiple human NSCLC lines such as A549, H460, H1299, H358, H2170, and Calu-1 (Figure 1C). Normal human lung cell line BEAS2B has a certain level of CD276, while 16HBE shows low expression. The overall expressions of CD276 in human NSCLC lines are higher than in normal lung tissue. The mouse NSCLC, LLC, and CMT167 lines have low and minimal (or no) expressions of CD276, respectively. These data are consistent with the reported high CD276 expression in multiple tumors or cancers in the literature [22]. Therefore, the human H358 and H460 were selected as CD276^+^ lines to evaluate the specific targeting and cytotoxicity in vitro and anti-NSCLC efficacy in vivo of the constructed ADC. The mouse LLC line was used to establish immunocompetent mouse models for the evaluation of synergetic anti-NSCLC efficacy in vivo.

### 3.2. NSCLC Surface Binding and In Vivo Targeting

A murine anti-human CD276 mAb was developed using hybridoma technology, produced in a Hybridoma serum-free medium in fed-batch shaker flask culture, and purified using a liquid chromatography system equipped with a protein A column following our previously established procedures [12,29,30,31,32,33,34]. The NSCLC targeting and drug delivery capability of the purified CD276 mAb were evaluated in vitro and in vivo.

**Figure 2:**
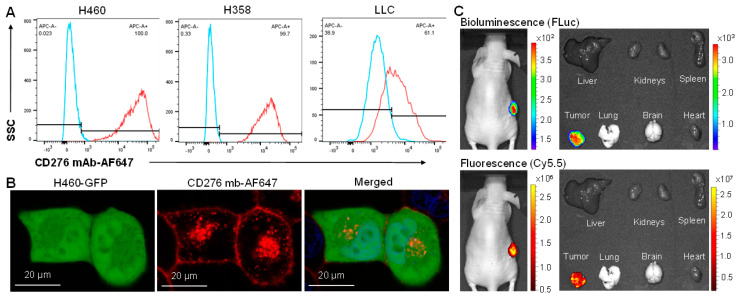
Evaluations of lung cancer surface binding, targeting, and delivery of CD276 mAb. (**A**) Analysis of NSCLC surface binding by anti-human CD276 mAb using flow cytometry. Staining conditions: 1 µg or 5 µg of anti-human CD276 mAb-AF647 per 1 × 10^6^ human NSCLC H460 and H358 cells or mouse LLC cells at room temperature for 30 min or 37 °C for 180 min, respectively. (**B**) Confocal microscope imaging showed strong surface binding and the internalization of CD276 mAb-AF647 (red) to H460 cells (green, infected with GFP virus) at 2–24 h after mixing. (**C**) IVIS imaging confirmed the in vivo NSCLC targeting of CD276 mAb-Cy5.5 in s.c. H460-FLuc xenografted nude mice. The live-animal images were collected at 24 h post-injection of Cy5.5-labelled mAb, followed by scarification, tumor and organ harvest, and ex vivo imaging.

First, the in vitro NSCLC surface binding of our murine anti-human CD276 mAb was evaluated in flow cytometry analysis using human NSCLC lines H460 and H358 and mouse NSCLC line LLC (Figure 2A). The murine mAb had a high surface binding rate to H460 (100%) and H358 (99.7%) but a lower binding rate to LLC (61.1%). The human CD276 (UniProt H0YL10) and mouse CD276 (UniProt A0A8C5XZR2) have a different sequence, topology, and molecular weight (90–110 kDa vs. 45–66 kDa). The murine anti-human CD276 mAb used in this study targets the conserved extracellular domain that has 93% similarity between human and mouse CD276 receptors. Therefore, the AF647 fluorescent dye labeled anti-human CD276 mAb (CD276 mAb-AF647) can target both human and mouse CD276 receptors, although it had higher surface binding to human NSCLC than mouse NSCLC, as confirmed in this flow cytometry analysis. These data also indicated that the NSCLC xenograft mouse models can be used to evaluate tumor targeting and treatment efficacy.

Second, live-cell confocal microscope imaging was performed to assess the NSCLC cell surface binding and drug delivery ability of CD276 mAb. As shown in Figure 2B, the H460 cells infected with BacMam GFP (Green) can be in vitro targeted by CD276 mAb-AF647 (red) within two hours, and the conjugated dye (or drug) can be intracellularly delivered after internalization of mAb within 24 h.

**Figure 3:**
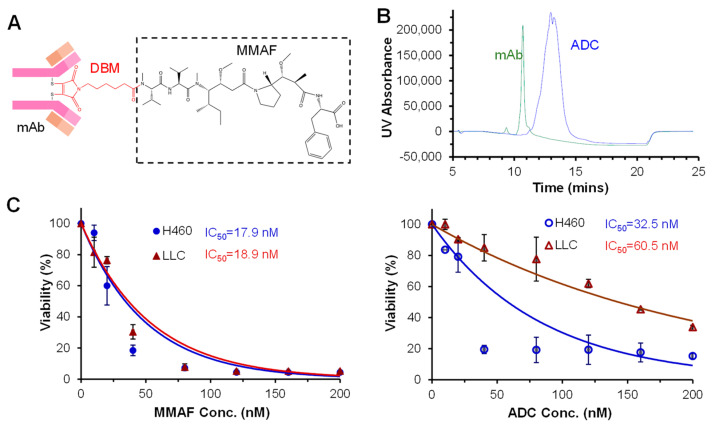
In vitro evaluations of free drug and ADC using both human and mouse NSCLC cell lines. (**A**) Structure of murine anti-human CD276 mAb carrying MMAF. (**B**) HPLC characterization of mAb and constructed ADC. (**C**) Cytotoxicity and IC_50_ of free MMAF and ADC. H460 cells were seeded in a 96-well plate at 3000 cells/well, and LLC cells were seeded at 1000 cells/well.

Third, the NSCLC tumor targeting was confirmed in H460-FLuc xenografted nude mice i.v. injected with Cy5.5 fluorescence-labeled CD276 mAb (Figure 3C). The live-animal IVIS imaging showed that CD276 mAb (indicated by fluorescence) could target and accumulate in NSCLC tumors (indicated by firefly bioluminescence) in vivo. The NSCLC-specific targeting was further confirmed with ex vivo IVIS imaging, which demonstrated a good overlap of Cy5.5 fluorescence and FLuc bioluminescence in the freshly harvested tumor, while there was no or an undetectable overlap of off-target mAb-Cy5.5 in major organs, including the brain, heart, lung, spleen, kidney, and liver. Altogether, the flow cytometry, confocal microscope, and IVIS imaging revealed that our murine anti-human CD276 mAb could specifically target NSCLC and effectively deliver a potent payload. These results indicated that CD276 mAb has great potential for the development of targeted therapy with high tumor specificity and minimal side effects, which could be translated to treat advanced NSCLC in clinics in the future.

### 3.3. Construction and Anti-NSCLC Cytotoxicity of ADC

Our cytotoxicity studies showed high potency and low cell viability of MMAF at the end of treatment using both human and mouse NSCLC cell lines (Figure 3C), so MMAF was selected as the chemotherapy for ADC construction. The purified CD276 mAb was conjugated with MMAF via re-bridging the DBM linker, which could maintain the integrity of mAb and drug–antibody ratio range of 3–4 using our established cysteine-based conjugation procedure with modification [29,31]. Briefly, 1 mg/mL of anti-CD276 mAb was reduced with 0.3 mM tris(2-carboxyethyl)phosphine hydrochloride (TCEP-HCl) at 37 °C for 30 min, and then reacted with a 10 mM re-bridging linker DBM and payload MMAF pre-conjugate (DBM-MMAF) stock at molar ratio of 1:7 or 1:10 at room temperature for 60 min in dark. The constructed CD276 mAb-MMAF (Figure 3A) was purified with a protein A column and/or G25 desalting column. The characterization of mAb-MMAF using a high-performance liquid chromatography (HPLC) system equipped with an MAbPac HIC-Butyl column indicated a high conjugation rate of 95–100%, a purity of >95%, and an integrity of ~100%. The HIC and UV/Vis analysis showed an average DAR of ~3.0–4.0, indicating the heterogeneous conjugation of the drug (Figure 3B).

The in vitro anti-NSCLC cytotoxicity of CD276 mAb-MMAF was tested in human H460 and mouse LLC lines with free MMAF as the control. ADC or MMAF at doses of 0, 10, 20, 40, 80, 120, 160, and 200 nM were tested in a 96-well plate-based 5-day MTT assay. The relative cell viabilities reached 4% (H460) and 5% (LLC) post-treatment with MMAF at titers of 120–200 nM, 15% (H460), and 34% (LLC) treated with 80–200 nM of ADC (Figure 3C). IC_50_ values of CD276 mAb-MMAF were 32.5 nM and 60.5 nM, and IC_50_ values of MMAF were 21 nM and 47 nM for human H460 and mouse LLC, respectively. These MTT assays showed that the cytotoxicity of ADC and free drugs was in the same range but the IC_50_ of ADC was a little higher than free drugs. The slightly reduced in vitro cytotoxicity of ADC could be caused by the fact that ADC increased the therapeutic specificity to NSCLC, but the surface binding, internalization, and drug release slowed down the process of drug release in vitro. The finding that LLC with faster cell growth and lower CD276 surface binding rate had a lower response to CD276 mAb-MMAF also explained the increased IC_50_ value.

### 3.4. Toxicity Evaluation of ADC

**Figure 4:**
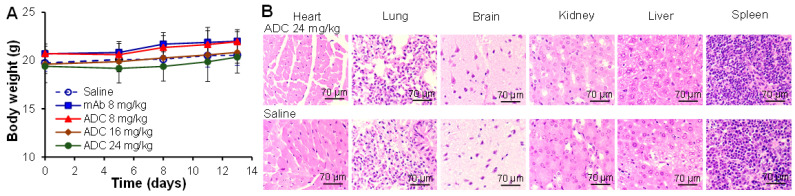
Toxicity study of ADC in C57BL mice with an i.v. injection of CD276 mAb-MMAF (8, 16, 24 mg/kg), CD276 mAb (8 mg/kg), or saline (control). Five groups, *n* = 6. (**A**) Body weight. (**B**) HE staining of the parafilm section of major organs (brain, heart, liver, kidney, lung, spleen) revealed no toxicity. Scale bar = 70 µm.

Three doses of anti-CD276 ADC (8, 16, and 24 mg/kg), CD276 mAb (8 mg/kg), and saline were injected into C57BL mice via tail vein (*n* = 6). The body weight of these mice had no obvious change at doses of 8–24 mg/kg ADC (Figure 4A). The general healthy status of mice, i.e., breath, locomotion, and water intake, had no difference among ADC, mAb, and saline groups. The H&E-stained parafilm sections of major organs (heart, lung, brain, kidney, liver, spleen) did not show any morphology change, necrosis, or damage, indicating no off-target and toxicity of ADC and mAb. In the future, we will evaluate higher ADC doses, such as 60–80 mg/kg, to achieve better anti-tumor efficacy and also identify the maximal tolerated dosage (MTD).

### 3.5. Anti-NSCLC Efficacy and Tumoral Immunity in LLC Xenograft Models

**Figure 5:**
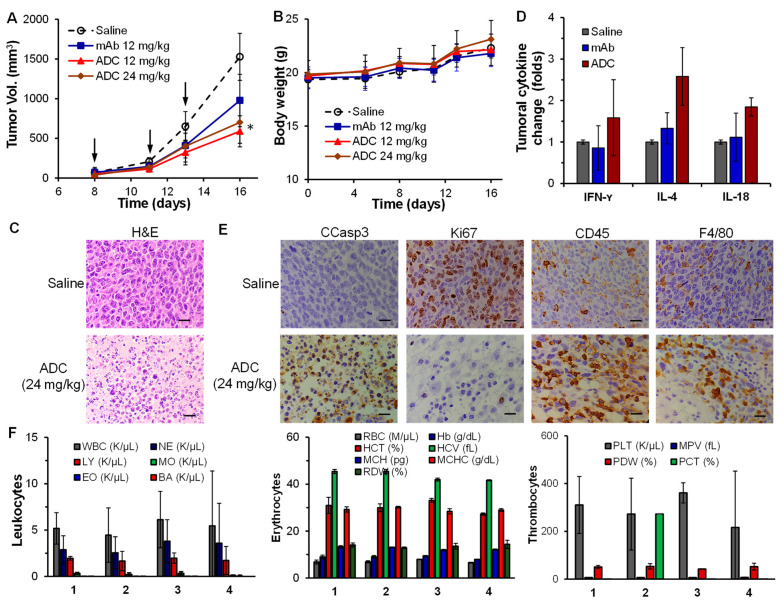
In vivo anti-NSCLC efficacy in LLC-FLuc allografted C57BL mouse models. (**A**) Tumor volume. Arrow indicated the i.v. injection of CD276 mAb-MMAF ADC (12 and 24 mg/kg), CD276 mAb (12 mg/kg, control), or saline (control) via tail vein. Tumor volume was measured with a caliper and calculated as an ellipsoid. Data were presented as mean ± SEM, five groups, *n* = 6. Data were presented as mean ± SEM. * *p* < 0.05 vs. saline using ANOVA followed by Dunnett’s *t*-test. Saline (○), 12 mg/kg of anti-CD276 mAb (■), 12 mg/kg of ADC (▲), and 24 mg/kg of ADC (♦). (**B**) Body weight. (**C**) HE staining of the tumor tissue slide post-treatment indicating severe tumor cell death in the ADC-treated group compared to the control group. Scale bar = 20 µm. (**D**) Luminex analysis of tumoral cytokine. *n* = 3. (**E**) IHC staining of tumor tissue with the Abs of cleaved caspase 3 (CCasp3, apoptosis), Ki67 (proliferation), CD45 (activated T/NK cells), and F4/80 (macrophage) demonstrated an increase in tumor cell proliferation, apoptosis, and the activation/infiltration of immune cells. Scale bar = 20 µm. (**F**) Whole blood analysis did not detect any toxicity of anti-CD276 mAb-MMAF. *n* = 2 or 3. 1: Saline; 2: CD276 mAb (12 mg/kg); 3: ADC (12 mg/kg); 4: ADC (24 mg/kg).

To evaluate the synergetic anti-NSCLC efficacy of CD276 mAb-MMAF, about 3 × 10^6^ mouse LLC-FLuc cells were s.c. injected into C57BL mice to establish immunocompetent models. The mice were i.v. injected with saline (vehicle, control), 12 mg/kg anti-CD276 mAb (control), and 12 and 24 mg/kg anti-CD276 mAb-MMAF (*n* = 6). As shown in Figure 5A, the NSCLC tumor growth rate was significantly reduced by 12 and 24 mg/kg ADC and 12 mg/kg mAb, with 61.5% and 26.1% tumor burden reduction, respectively, compared to the control group. These data indicated that the integration of cancer cell proliferation inhibiting payload (MMAF) and the immune checkpoint blocking mAb (anti-CD276 mAb) could effectively inhibit NSCLC tumor growth. Further optimization of ADC doses in future anti-NSCLC efficacy studies could achieve better tumor burden reduction. Similar body weight profiles among the control and treatment groups suggested no or minimal toxicity of ADC treatment (Figure 5B). The H&E staining of tumor tissues post-treatment (Figure 5C) revealed significant tumor cell death in the 24 mg/kg anti-CD276 mAb-MMAF treatment group, while tumor cells were healthy in the saline group. The reduced tumor growth and severe cell death suggested the great potential of CD276-targeted ADC for NSCLC treatment, which will be fully investigated in future treatment strategy (dose and schedule) optimization and survival studies. We can also consider cleavable linkers and other potent payloads to achieve higher anti-tumor efficacy.

To understand the anti-NSCLC mechanisms, we first performed a Luminex assay using the tumor tissues harvested at the end of treatment to titrate the cytokine secretion in the tumor microenvironment. As presented in Figure 5D, the IFN-γ, IL-4, and IL-8 concentrations were slightly increased by anti-CD276 mAb but significantly increased by 58%, 158%, and 85% with ADC treatment, indicating the reactivation of infiltrated T/NK cells. Second, the IHC staining of parafilm sectioned tumor slides showed that ADC treatment caused obvious apoptosis (indicated by an increase in CCasp3 Ab staining), reduced cell proliferation (indicated by a reduction in the positive staining of Ki67 Ab), activated T/NK cells (indicated by enhancement of positive staining of CD45 Ab), and improved macrophage infiltration (indicated by higher F4/80 Ab staining). Both H&E and IHC staining of ADC-treated tumor tissues showed reduced cell density, which is consistent with the tumor volume results in Figure 5A.

In addition to NSCLC treatment, we also tested the general toxicity in peripheral blood two weeks after ADC treatment by performing whole blood analysis with HemaVet 950FS. As summarized in Figure 5F, complete blood count (CBC) of blood samples collected through cardiac puncture did not show an obvious difference in the cell count of leukocytes (white blood cell, neutrophil, lymphocyte, monocyte, eosinophils, basophile), erythrocytes (red blood cell, hemoglobin, hematocrit), and thrombocytes (platelet, plateletcrit) among different groups.

All these data demonstrated that our anti-CD276 mAb-MMAF can effectively reduce NSCLC tumor burden via inhibiting microtubule polymerization by MMAF, reactivating immune cells (T, NK, macrophage), increasing immune cells infiltration, and/or enhancing the secretion of cytokines in the tumor. The anti-mouse NSCLC efficacy could be further improved by optimizing the ADC treatment schedule and dosage. Importantly, the off-target toxicity or side effect was minimal, as confirmed in both the H&E staining of major organs and whole blood analysis, suggesting that our anti-CD276 ADC is a promising targeted therapy for NSCLC treatment.

### 3.6. Anti-NSCLC Efficacy Validation in H460 Xenograft Models

**Figure 6:**
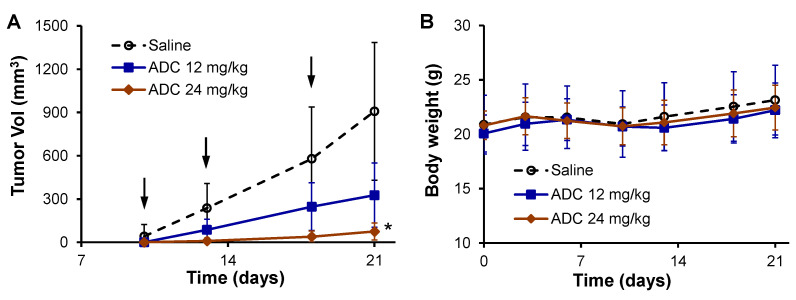
In vivo anti-tumor efficacy in human NSCLC xenografted nude mouse models. The H460-FLuc xenografted nude mice were treated with ADC (12 and 16 mg/kg CD276 mAb-MMAF) or saline (control) via i.v. injection through the tail vein. (**A**) Tumor volume post-treatment is indicated by a black arrow. Tumor volume was measured with calipers and calculated as an ellipsoid. Data were presented as mean ± SEM. *n* = 5. * *p* < 0.05 vs. saline. Saline (○), 12 mg/kg of anti-CD276 mAb (■), and 24 mg/kg of ADC (♦). (**B**) Profiles of body weight.

Human NSCLC xenografted models were established by s.c. implanting 3 × 10^6^ H460-FLuc cells into nude mice. As described in Figure 6A, the 12 and 24 mg/kg ADC treatment reduced tumor growth by 64.1% and 91.9%, respectively, compared to the saline group. The body weight monitored twice a week did not show a significant difference between the control and treatment groups (Figure 6B). The better treatment efficacy of the human NSCLC tumor could be explained by the higher targeting and binding rate of anti-human CD276 mAb to the human surface receptor than the mouse surface receptor, as detected in flow cytometry (100.0% vs. 61.1%) and the higher drug delivery efficacy in vivo.

An antibody-drug conjugate has been investigated and widely used as an effective targeted therapy to treat various cancers [12,29,31,32,33]. Treating NSCLC with ADC has been actively investigated recently due to the advantages of cancer-specific targeting by mAb, high potency of the delivered small-molecule therapy, minimal toxicity to normal organs and tissues, high circulation stability, and potential tumoral immunity of mAb [35,36,37,38,39]. Multiple mAbs and ADC-based targeted therapies have been approved by the FDA for NSCLC treatment, including trastuzumab-deruxtecan, EGFR/MET bispecific amivantamab, atezolizumab, bevacizumab, durvalumab, necitumumab, nivolumab, pembrolizumab, ramucirumab, tremelimumab-actl, and ipilimumab.

To our knowledge, the CD276-targeting therapy (mAb or ADC) has not been developed for NSCLC treatment so far. As reported in this study, CD276 is a promising surface receptor to target NSCLC due to its high patient coverage rate (over 70%), despite the genome background or driving mutations. Our anti-human CD276 mAb has high specificity to CD276^+^ NSCLC and exhibits strong cross-activity to both human and mouse CD276. Therefore, the cancer treatment evaluation using xenograft mouse models is translational. The constructed ADC (i.e., CD276 mAb-MMAF) demonstrated high anti-tumor efficacy in both in vitro studies using multiple cell lines and in vivo studies using two animal models. The post-treatment analysis in immunocompetent models indicated strong synergetic anti-NSCLC mechanisms of our ADC, including direct tumor cell death by MMAF, which has been used to treat other cancers, immune responses, such as cytokine secretion and reactivation in the tumor microenvironment, and the blockage of the CD276 immune checkpoint, which needs further evaluation. The synergism of these cancer treatment mechanisms resulted in significant tumor growth inhibition (up to 92%) in xenograft models. Moreover, the toxicity studies did not detect any damage, inflammation, or other off-target side effects in major organs, immune toxicity in peripheral blood, adverse body weight changes, or behavior changes at a dose of 24 mg/kg. Taken together, the anti-CD276 ADC developed in this study could specifically target CD276-positive NSCLC cell lines and xenografts and deliver potent small molecules to effectively treat NSCLC with minimal side effects.

To translate the developed CD276-targeting ADC for NSCLC patient treatment in the future, we need further development and evaluation to provide guidance for future preclinical or clinical studies. For instance, we need to construct chimeric and humanized CD276 mAb to increase its half-life and plasma stability; evaluate and conjugate higher potent drugs with lower IC_50_ values (e.g., MMAE and DM1) using new linkers (e.g., cleavable linker and others); investigate tumoral immunity in patient-derived xenograft humanized mouse models; and/or optimize ADC dosage by testing a high dosage of 60–80 mg/kg and treatment schedules. Combining the developed CD276-targeted ADC with other therapies could further improve NSCLC treatment efficacy.

## 4. Conclusions

This study reported a new antibody-drug conjugate to treat NSCLC by integrating multiple anti-cancer mechanisms. Both in vitro and in vivo evaluations indicated that our anti-CD276 ADC has great potential to target and effectively treat CD276 overexpressing NSCLC with undetectable side effects.

## Data Availability

The data presented in this study are contained within the article.
